# Completion of community health worker initiated patient referrals in integrated community case management in rural Uganda

**DOI:** 10.1186/s12936-018-2525-9

**Published:** 2018-10-22

**Authors:** Jana Jarolimova, Stephen Baguma, Palka Patel, Sara Mian-McCarthy, Moses Ntaro, Michael Matte, Jessica Kenney, Shem Bwambale, Edgar Mulogo, Geren Stone

**Affiliations:** 1 Global Health Collaborative, Mbarara, Uganda; 2 Department of Medicine, Massachusetts General Hospital, 55 Fruit St, Boston, MA 02114 USA; 3 Bugoye Health Center, Bugoye, Uganda; 4 Mbarara University of Science and Technology, P.O. Box 1410, Mbarara, Uganda; 5 Massachusetts General Hospital Center for Global Health, 125 Nashua Street, Suite 722, Boston, MA 02114 USA

**Keywords:** Integrated community case management, Community health worker, Patient referral, Referral completion, Monitoring and evaluation

## Abstract

**Background:**

Uganda has sought to address leading causes of childhood mortality: malaria, pneumonia and diarrhoea, through integrated community case management (iCCM). The success of this approach relies on community health worker (CHW) assessment and referral of sick children to a nearby health centre. This study aimed to determine rates of referral completion in an iCCM programme in rural Uganda.

**Methods:**

This was a prospective observational study of referrals made by CHWs in 8 villages in rural western Uganda. All patient referrals by CHWs were tracked and health centre registers were reviewed for documentation of completed referrals. Caregivers of referred patients were invited to complete a survey 2–3 weeks after the referral with questions on the CHW visit, referral completion, and the patient’s clinical condition.

**Results:**

Among 143 total referrals, 136 (94%) caregivers completed the follow-up survey. Reasons for visiting the CHW were fever/malaria in 111 (82%) cases, cough in 61 (45%) cases, and fast/difficult breathing in 25 (18%) cases. Overall, 121 (89%) caregivers reported taking the referred child for further medical evaluation, of whom 102 (75% overall) were taken to the local public health centre. Ninety per cent of reported referral visits were confirmed in health centre documentation. For the 34 caregivers who did not complete referral at the local health centre, the most common reasons were improvement in child’s health, lack of time, ease of going elsewhere, and needing to care for other children. Referrals were slightly more likely to be completed on weekdays versus weekends (p = 0.0377); referral completion was otherwise not associated with child’s age or gender, caregiver age, or caregiver relationship to child. One village had a lower rate of referral completion than the others. Improvement in the child’s health was not associated with completed referral or timing of the referral visit.

**Conclusions:**

A high percentage of children referred to the health centre through iCCM in rural Uganda completed the referral. Barriers to referral completion included improvement in the child’s health, time and distance. Interestingly, referral completion at the health centre was not associated with improvement in the child’s health. Barriers to referral completion and clinical management at all stages of referral linkages warrant further study.

## Background

International focus on universal health care and the Sustainable Development Goals has led to a resurgence in utilization of community health workers (CHWs) in low and middle income countries [1], including employment of CHWs in efforts to curb child mortality. CHWs have been shown to appropriately manage common childhood illnesses, including malaria [2–4], diarrhoeal illness and pneumonia [5–8], and have reduced morbidity and mortality with community-based management of pneumonia and malaria [9, 10]. In 2012, the WHO and UNICEF formally endorsed Integrated Community Case Management (iCCM), an evidence-based algorithmic management approach employed by CHWs, to jointly address the leading causes of under-5 mortality (malaria, pneumonia, diarrhoea [11]), and by 2013, 28 sub-Saharan African (SSA) countries had implemented iCCM [12]. iCCM involves diagnostic and therapeutic services in the community and relies on referral linkages from CHWs to health facilities.

Despite improvements in child health over the last two decades [13, 14], Uganda continues to have an under-5 mortality rate of 64 per 1000 live births [14]. In 2010, the Ugandan Ministry of Health (MOH) introduced iCCM on a national level [15]. In the initial Ugandan model, groups of 4–5 CHWs per village, each responsible for 25–30 households, were trained to provide a number of services, including preventive health care, community-based health education and iCCM. For iCCM, CHWs use an algorithmic protocol for assessment of a sick child, including a targeted physical examination, history and a rapid diagnostic test (RDT) for malaria for children presenting with fever based on history or examination. Based on the results, CHWs provide treatment in the community or, for particular conditions or danger signs, refer the patient to a nearby public health facility.

Patient referrals are a critical component of the iCCM care framework [12], ensuring that patients who cannot be safely managed in the community are evaluated at a nearby health facility. Reducing under-5 mortality in the iCCM model depends on the completion of these referrals and further evaluation and treatment of these children who may be the most ill. Rates of reported referral completion have varied widely in CHW programs worldwide [16–21], from 1.5% in a study of RDT-negative patients in a malaria treatment programme in Sierra Leone [16] to 93% for a Home Based Management of Fever (HBM) programme in Uganda [21]. A previous iCCM-specific evaluation from Uganda demonstrated a referral completion rate of 45.7% [19]. Efforts to obtain accurate completion rates in these studies have been hampered by recall bias and poor health facility documentation.

Obtaining a better understanding of factors affecting referral completion is a global research priority for iCCM [22]. In order to evaluate factors affecting successful referral completion in an iCCM programme, there must first be a reliable method for accurate measurement of referral completion rate. This study utilized a novel, prospective approach for determination of the true rate of referral completion and determinants of completed referrals within an iCCM programme in rural western Uganda.

## Methods

### Study site and population

The Bugoye Integrated Community Case Management Initiative (BIMI) is a collaboration between Bugoye Health Centre III (BHC III), Mbarara University of Science and Technology (MUST), and Massachusetts General Hospital (MGH). Established in 2011, the partnership aims to improve the health of the community in Bugoye, a rural sub-county in western Uganda, while strengthening the local health system and innovating in community-based care. At the time of this study, the BIMI programme was operated by 8 Ugandan staff members (4 full-time, 3 part-time and one research assistant), and 3 part-time staff members at MGH. The BIMI programme has trained 38 individual CHWs in 8 villages in iCCM, and provides the CHWs with regular supervision, refresher training and medication supplies. The BIMI iCCM programme operates in an area of high malaria incidence; since programme inception, 73.4% of children evaluated by BIMI CHWs for fever have had a positive RDT result. According to a household census in 2016, the 8 villages in which BIMI operates comprised approximately 1117 households with a total of 1237 children under 5 years old.

Caregivers of children under 5 seeking community-based care for acute illness from a BIMI CHW, who were referred to the health centre as part of iCCM management, were eligible to participate. Caregivers younger than 18 years old were excluded.

### Intervention

All referrals made by the CHWs during the study period were reported in real time via Short Message Service (SMS) messaging to BIMI programme staff. There is a high rate of mobile phone ownership in the area where this study was conducted. CHWs were provided with a small monthly mobile airtime stipend (equivalent at the time of $0.67/CHW/month) to offset the cost of study-related SMS messages. CHWs were then contacted by phone and information on the referred patient, caregiver and time of referral was recorded. A trained research assistant reviewed outpatient and inpatient clinical registers at BHC III for documentation of an outpatient visit or inpatient admission for the referred patient. Records were matched based on patient name, date of birth/age and village. Based on health centre registers, referrals were recorded as completed ‘within 24 h’, ‘between 1 and 7 days’, or ‘not at all’.

Two to three weeks after the referral date, a trained research assistant fluent in the local language (Lhukonzo) visited the caregiver of the child at home to administer a one-time survey. The caregiver was notified by phone in advance and attempts in person were made to locate the caregiver. If the caregiver did not own a mobile phone, contact was made through the referring CHW. If unable to locate the caregiver after three attempts, the questionnaire was marked as not completed. Priority was given to an interview of the person who had taken the child to see the CHW; if this caregiver was younger than 18 years old and not the primary caregiver, then the primary caregiver was identified and interviewed if over the age of 18. Informed consent was obtained in Lhukonzo from all participants. The questionnaire asked about demographics of the caregiver and child, details of the child’s acute illness and CHW visit, and questions on the timing, location and experience of up to three referral visits, if one or more were completed. If no referral visit to BHC III was made, the caregiver was asked about barriers to referral. All caregivers were asked if the child’s health had improved; children who had not improved were referred immediately to the health centre. The study was conducted from 1 April, 2017 to 31 December, 2017.

### Measures

The primary outcomes of interest were self-report of a completed referral visit to BHC III, completed referral visit to a different health care provider (formal or informal), and improvement in the child’s health. BHC III clinic documentation was examined in order to determine the proportion of reported completed referrals that could be confirmed at the clinic. Exposures of interest for referral completion were determined from prior literature and based on discussion with local health care providers and CHWs. These included village, referral day of week, child age, child gender, caregiver age, and caregiver relationship to child. Exposures of interest for improvement in child’s health included referral completion at BHC III, completion of referral to another site, and time to first referral visit. Data for all exposures were obtained from the questionnaire.

### Statistical analysis

Descriptive statistics were used to summarize the characteristics of patients and caregivers, perceived reasons for referral, referral completion and barriers to referral completion. Associations between exposures and outcomes were tested using Chi square or Fisher’s Exact test for categorical variables, and t-test or Wilcoxon Rank Sum test for continuous variables. Logistic regression was used to test for effect of ordinal and non-binary categorical predictors on outcomes.

### Ethical approval

This study was approved by the Ethics Review Boards of the Mbarara University of Science and Technology and Partners Healthcare. Administrative approval was secured from the Ugandan National Council for Science and Technology.

## Results

**Fig. 1:**
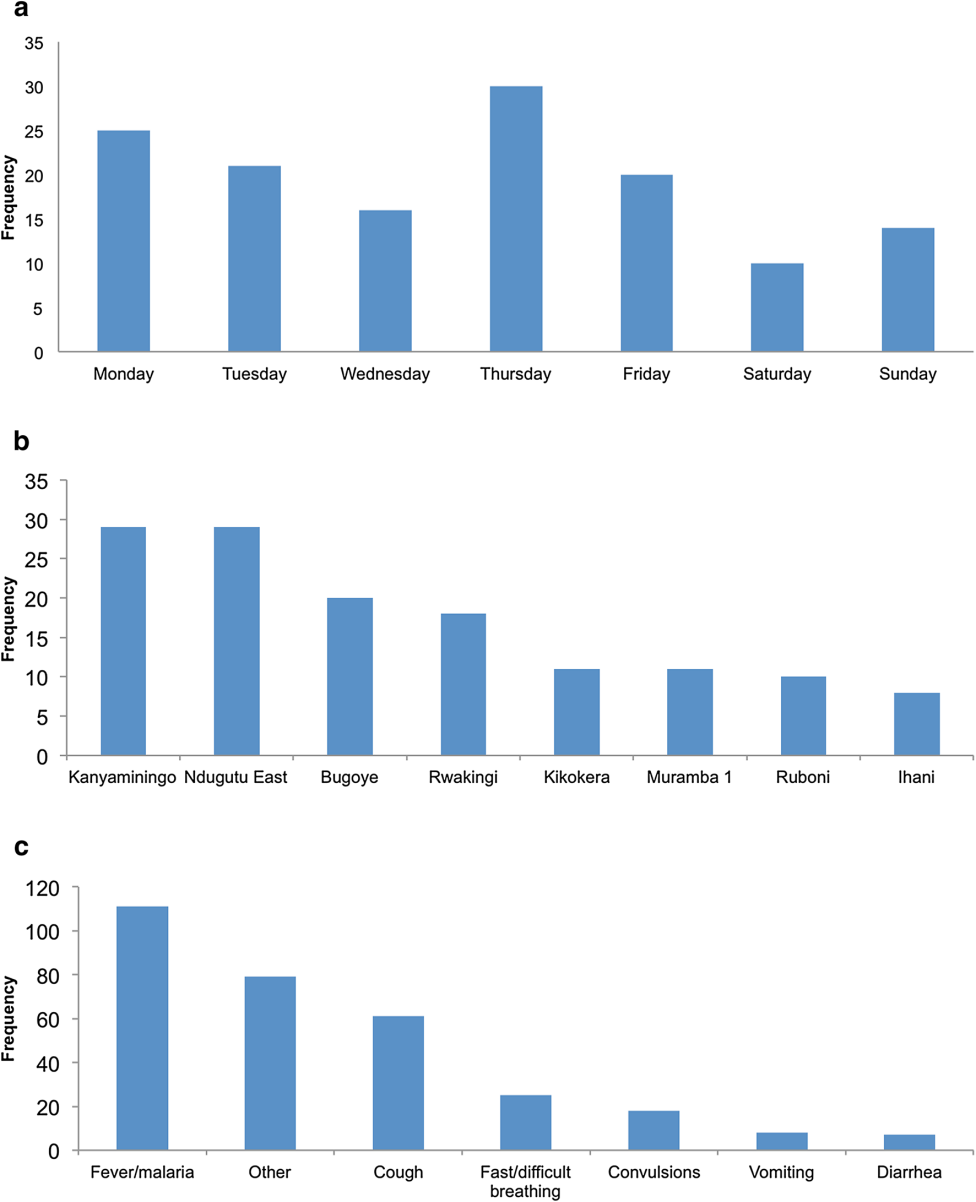
Characteristics of referrals (n = 136). Caregivers could list more than one reason for taking child to see the CHW

CHWs reported 143 referrals from the 8 programme villages from 1 April, 2017 to 31 December, 2017, among whom 136 caregivers of referred children met the inclusion criteria for the study and completed the questionnaire (3 caregivers were under 18 years of age, and 4 were not located). Demographics of patients and caregivers are summarized in Table 1. Patients were a median age of 1.69 years (20 months) and 52% female. Caregivers interviewed were a median 26 years old and 123 (90%) were biological mothers of the patient. Five (3.7%) of the caregivers reported *not* understanding why the child was referred to the health centre. Characteristics of the 136 referrals are represented in Fig. 1. All 8 participating villages, and 28 CHWs (among 35 total CHWs supported by the BIMI programme) were represented. Referrals were most frequently made on Mondays and Thursdays, and least commonly on Saturdays and Sundays (p = 0.0276 for test of equal proportions). The most frequent reasons for taking the child to see the CHW were fever/malaria (n = 111, 82%), cough (n = 61, 45%), and fast/difficult breathing (n = 25, 18%).

**Table 1:** Characteristics of referred patients and their caregivers, n = 136

Caretaker age (years), Median [IQR]	26.0 [22, 30]
Child age (years), Median [IQR]	1.69 [0.69, 3.02]
Child gender	n (%)
Male	65 (48%)
Female	71 (52%)
Primary caregiver relationship to child	
Biological mother	123 (90.5%)
Biological grandmother	7 (5.2%)
Biological father	1 (0.7%)
Biological aunt	1 (0.7%)
Other	3 (2.2%)
Missing	1 (0.7%)
Caregiver understood why referral was made	
Yes	131 (96.3%)
No	5 (3.7%)

**Fig. 2:**
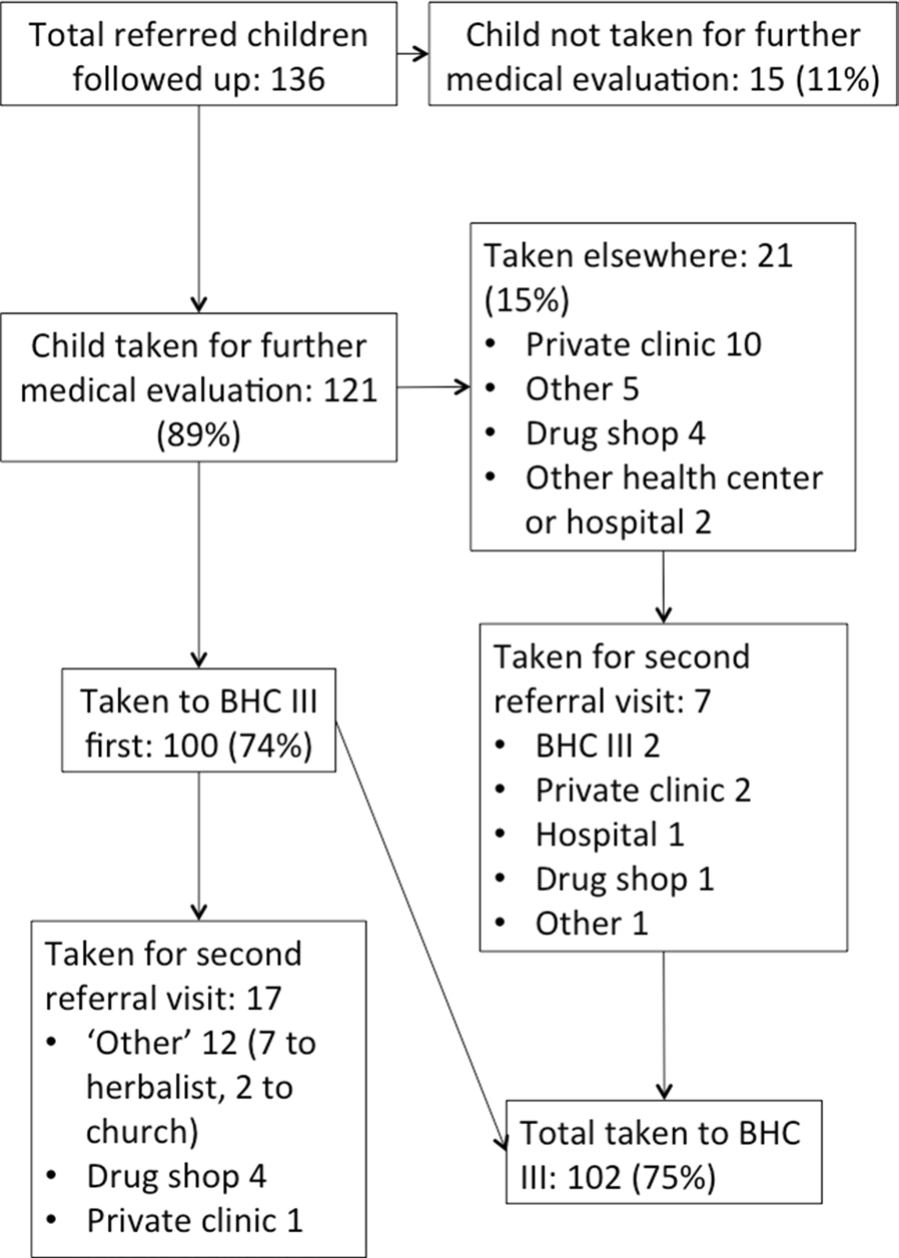
Overall referral completion at BHC III and other sites as reported by caregivers of referred patients

Among all referred patients, 121 (89%) were taken for further evaluation and treatment, with 100 of these taken to BHC III for the first referral visit, and 102 (75% of all referrals) taken to BHC III for any referral visit. Other referral visit sites are summarized in Fig. 2. No caregivers reported taking the child to a traditional healer or a different CHW for the first referral visit. Ninety-seven of 121 referrals (71%) were reported as completed on the same day, and 17 (12.5%) on the following day. Of the 121 children who were taken for a referral visit, 27 (22%) were taken for a second referral visit to a different location. Seventeen (17%) of the 100 children initially taken to BHC III were later taken for a second referral visit, including 4 who were taken to a ‘drug shop’ (private pharmacy), and 12 taken to ‘other’ locations, including 7 visits to a ‘herbalist’ and 2 to a church. Two patients—one who was initially taken to a drug shop and one to a private clinic—were subsequently taken to BHC III, with a total of 102 out of 136 patients (75%) taken to BHC III for the first or second referral visit. Review of clinical registers at BHC III, initially done prospectively but additionally repeated retrospectively to clarify discrepancies in patient information, revealed 92 (90%) of 102 reported referrals documented as completed at the health centre.

**Fig. 3:**
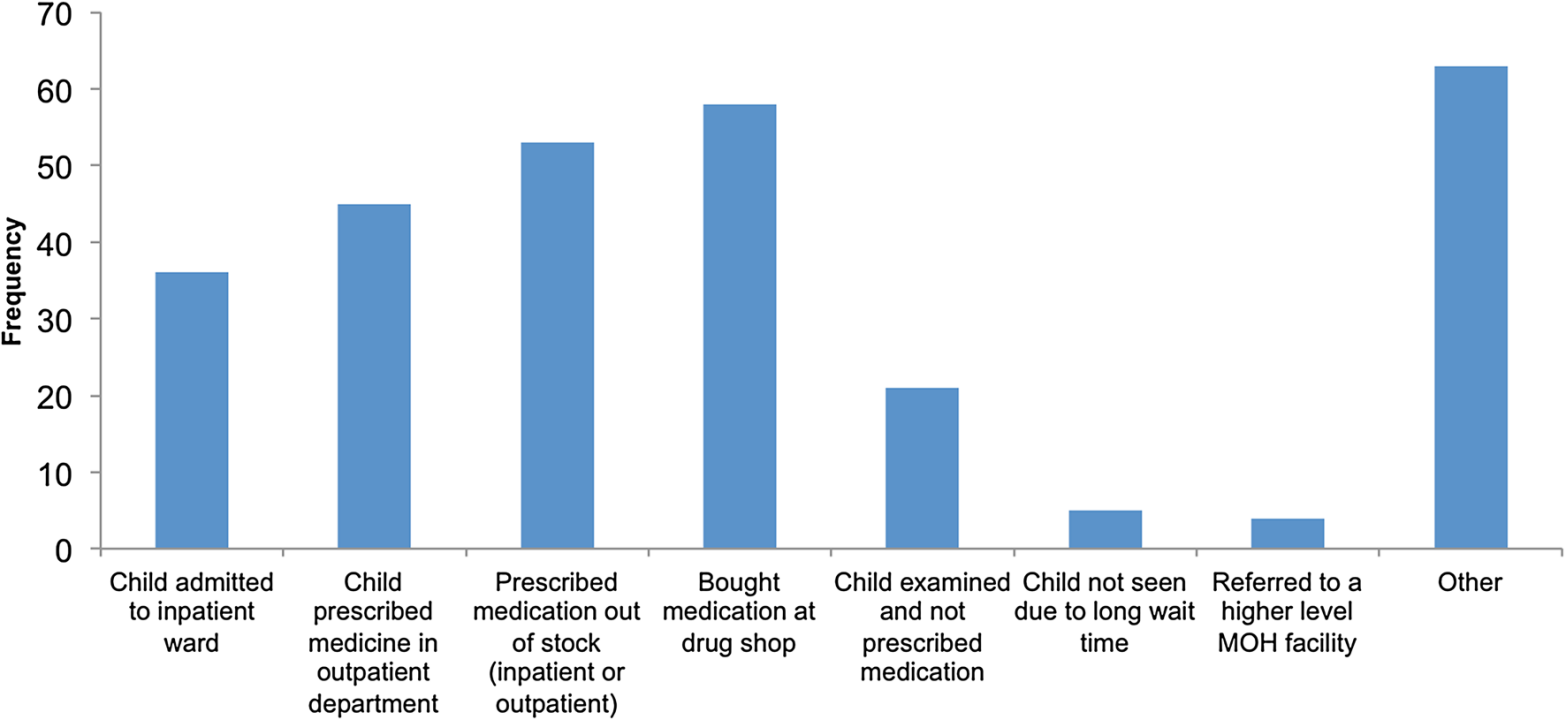
Reported experiences of the referral visit. Caregivers could answer in more than one category

Reported experiences at the health centre are shown in Fig. 3. Four patients were referred to a higher-level MOH facility, of whom 2 (50%) completed this higher facility referral.

**Fig. 4:**
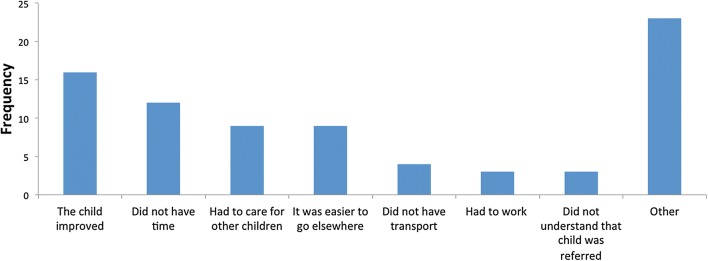
Barriers to referral completion among those who did not complete referral visit at BHC III. Caregivers could answer in more than one category

The most frequent reasons cited by the 34 caregivers who reported not taking their child to BHC III for a referral visit included that the child’s health improved, caregiver’s lack of time, perception that it was easier to go elsewhere, or need to care for other children (Fig. 4). ‘Other’ reasons included concerns about lack of medication supply at BHC III, and belief that the health centre would not be open, primarily on holidays and weekends.

Association between patient, caregiver, and referral characteristics and completion of referrals at BHC III are shown in Table 2. Referrals were most likely to be completed on Tuesdays, and least likely to be completed on Mondays and Saturdays. They were also less likely to be completed on weekends (Saturday or Sunday) than on weekdays (p = 0.0377). Village 7 had a significantly lower rate of referral visit completion at BHC III (p = 0.0014), with only 33% of referrals completed; there were no significant differences in referral completion among the other villages. Completion of referral visit at BHC III was not associated with child gender, child age, caregiver age, or caregiver relationship to child.

**Table 2:** Association of patient, caregiver and referral characteristics with referral completion at BHC III, n = 136

	Completed referral at BHC III	Did not complete referral at BHC III	p-value
Caregiver age (years), median [IQR]	25 [22–30]	27 [22–32]	0.2013
Child age (years), median [IQR]	1.37 [0.60–3.0]	1.88 [1.02–3.04]	0.1353
Child gender, n (%)			0.6202
Male	50 (77%)	15 (23%)	
Female	52 (73%)	19 (27%)	
Primary caregiver relationship to child (n = 135)			
Biological mother	96 (78%)	27 (22%)	
Biological grandmother	4 (57%)	3 (43%)	0.2168^a^
Biological father	1 (100%)	0 (0%)	0.9889
Biological aunt	0 (0%)	1 (100%)	0.9887
Other	0 (0%)	3 (100%)	0.9805
Village			
1	19 (95%)	1 (5%)	
2	5 (63%)	3 (37%)	0.0533^b^
3	20 (69%)	9 (31%)	0.0514
4	9 (82%)	2 (18%)	0.2642
5	9 (82%)	2 (18%)	0.2643
6	26 (90%)	3 (10%)	0.5108
7*	6 (33%)	12 (66%)	*0.0014*
8	8 (80%)	2 (20%)	0.2290
Day of week			
Monday*	15 (60%)	10 (40%)	0.0295^c^
Tuesday	19 (90%)	2 (10%)	
Wednesday	14 (88%)	2 (12%)	0.7737
Thursday	23 (77%)	7 (23%)	0.2170
Friday	17 (85%)	3 (15%)	0.5954
Saturday	5 (50%)	5 (50%)	0.0211
Sunday	9 (65%)	5 (35%)	0.0735
Weekday vs weekend*			*0.0377*
Weekday	88 (79%)	24 (21%)	
Weekend	14 (58%)	10 (42%)	

Univariate analyses calculated using t-test for continuous variables, Chi squared or Fisher’s exact test for binary variables, and logistic regression for non-binary categorical variables

* Statistically significant at p < 0.05

^a^Reference: Biological mother

^b^Reference: Village 1

^c^Reference: Tuesday

Of note, completion of a referral visit was not associated with caregiver-reported improvement in child health, whether the visit was completed at BHC III (p = 0.98) or anywhere (p = 0.55). Timing of referral visit to BHC III (in days from referral) was not associated with improvement in child health (p = 0.53).

## Discussion

Overall, a high percentage of caregivers of children referred to the health centre in this iCCM programme reported completing the referral. The majority of reported referrals were identified in health centre documentation, corroborating participant self-report. At 75%, the rate of completed referrals to the health centre determined by this prospective follow-up method is higher than that previously documented through the standard system of referral forms within the BIMI programme [23] and higher than that reported previously from an iCCM programme in Uganda [19]. Among those who brought the patient to BHC III, 96% reported making the referral visit within one calendar day of the referral (same day or next day), indicating high adherence to recommended referral timing.

Previous attempts to determine referral completion rates in CHW and iCCM programmes have been complicated by inaccurate measurement methods. Prior studies have primarily relied on retrospective review of health centre records to determine the number of completed referrals [16, 20], or by direct interview with caregivers of referred patients [18, 19, 21]. The retrospective nature of several of these studies may have led to recall bias, and lowered yields of referral cases found in clinical documentation. In iCCM programmes specifically, the standard method of referral documentation has relied on collection of paper-based referral forms provided by CHWs to caregivers. Prior studies of referral linkages in sub-Saharan Africa [24] as well as previous assessments within the BIMI programme [23] revealed either low or unknown numbers of referral forms brought to and collected at the health centre. This study introduced a novel method of real-time prospective referral tracking with individual short-term follow-up, which has not been previously described.

Referral completion in this study was not associated with child age or gender, or caregiver age. However, it overall appears that other systemic or logistical factors are playing a significant role in referral completion, such as distance to the health centre or day of the week. In this study, referrals were less likely to be completed on a weekend (Saturday or Sunday) than on a weekday. Consistent with this finding, on the questionnaire several caregivers voiced concerns that the health centre would not be adequately staffed on weekends or holidays. Distance from the health centre was expected to play a significant role in decisions affecting referral completion, as nearly 40% of residents of the 8 programme villages who identify BHC III as their nearest health centre report a travel time of 1 h or more to BHC III. However, the only village in this study (village 7) with a significantly lower referral completion rate than the other villages is one of the nearest to the health centre. Interestingly, this village is located along the main access road to the health centre and thus has easy transport access to the health centre, however this main access road also leads to a larger town and a private health centre in the other direction, thus residents of this village may decide to access these other referral sites more frequently.

Caregivers reported that approximately one-third of patients referred to the health centre were admitted to the inpatient ward and one-fifth were examined and sent home without a new medication; the remainder were prescribed a new medication in the outpatient clinic. These results suggest variability either in the severity of illness of the referred children, or their clinical management at the health centre. The majority of caregivers reported having to buy medication at a drug shop; similarly, caregivers who did not complete referrals often cited the expectation that medications would be out of stock at the health centre. Despite counselling by CHWs to take the child to BHC III, sick children were often taken to other locations for referral, most frequently private clinics and private pharmacies. Additionally, 7 of the 17 patients who were taken to a second referral visit after BHC III were taken to herbalists, suggesting a potentially significant role for these informal health care providers in the communities served by the BIMI programme.

Surprisingly, this study found no association between completed referral at the local health centre and reported improvement in the child’s health (Table 3). This was true both for referrals completed at BHC III, and for those completed at any location. While the sample size may have provided low power to detect a difference, this finding may be highlighting the need to ensure health centres such as BHC III are appropriately staffed and equipped to treat those children who are more severely ill. Alternatively, the iCCM algorithm may be indicating referral to the health facility for children who would otherwise have improved with conservative management. Finally, it is possible that caregivers who perceived their children as less seriously ill were less likely to take their children to the health centre, thus these children were more likely to improve despite not completing a referral visit. In the future it would be informative to ask caregivers about their perception of the child’s severity of illness at the time of referral.

**Table 3:** Association of referral completion and timing with improvement in child’s health

	Child health improved	Child health did not improve	p-value
Referral completion at BHC III, n = 135			0.9798
Yes	80 (79%)	21 (21%)	
No	27 (79%)	7 (21%)	
Referral completion at any location, n = 135			0.5482
Yes	96 (80%)	24 (20%)	
No	11 (73%)	4 (27%)	
Referral time among those who went to BHC III, by day from date of referral, n = 99	0.5345		

Univariate analyses calculated using Chi squared or Fisher’s exact test for binary variables, and logistic regression for ordinal variables

In sum, these data show a high rate of completed, timely referrals in an iCCM programme in rural Uganda, utilizing real-time tracking of referrals and short-term follow-up with caregivers. The results demonstrate the need to innovate around best approaches to documenting and reporting of completed referrals in iCCM programmes, and could extend more broadly to other community health worker programmes in rural areas in low and middle-income countries. This model of real-time referral tracking did not require significant human and financial resources, as SMS messages were a cost-effective means of CHW communication, and all follow-up caregiver interviews were carried out by one research assistant. Efforts to improve referral rates even further should focus on systemic barriers, such as means of transport, missing time from work, or negative perceptions about health centre staffing and drug stocks. Public education regarding the value of clinician review at the health centre, as well as continued support for the medication supply chain, will therefore be integral to improving referral rates further in the future. Finally, better understanding of the approach of non-traditional health care providers such as herbalists or other traditional healers to child illness should be pursued.

This study has several limitations. First, a relatively small sample size limits the power of the study for detecting minor differences in referral completion rates based on patient and caregiver demographics. Second, there may have been unintended consequences from implementation of the study protocol, specifically asking CHWs to report referrals made in real-time by SMS may have increased the motivation of CHWs and caregivers to ensure referral completion. Similarly, frequent review of health centre registers may have motivated health centre staff to be more thorough with recording referred patients, and thus may not reflect true referral documentation outside of the study period. However, this study provides a straightforward and effective framework for evaluation of accurate referral completion rates in other iCCM and CHW programmes.

## Conclusions

Understanding true rates of completed referrals within an iCCM programme provides an important opportunity to evaluate the efficacy of the referral network in a community-based approach to child illness and mortality. This study revealed a higher rate of both self-reported and documented referral completion than previously determined in an iCCM programme in rural Uganda. The approach employed demonstrates that real-time tracking and follow-up of referrals provides accurate monitoring and evaluation data for iCCM programmes, suggesting the need for innovation in referral tracking methods in practice. The connection between referral completion and improvement in child health in ICCM programmes warrants further study.

## Abbreviations

iCCM: Integrated Community Case Management
BIMI: Bugoye Integrated Community Case Management Initiative
HBM: Home-based Management of Fever
BHC III: Bugoye Health Center level III
MUST: Mbarara University of Science and Technology
CHW: community health worker
MOH: Ministry of Health
RDT: rapid diagnostic test
